# Notes and News

**Published:** 1900-02-24

**Authors:** 


### NOTES AND NEWS.

### HOSPITAL MEETINGS.

Seamen's Hospital.—Annual Meeting at four p.m., on Friday, February 23rd, at the Institute of Chartered Accountants, Moorgate Place

Great Northern Central Hospital—.Annual Meeting at five p.m., on Friday, February 23rd, at the Hospital.

Tottenham Hospital.—Annual Meeting at a quarter past five p.m., on Tuesday, February 27th, at the Hospital.

Cancer Hospital.—Annual Meeting at four p.m., on Wednesday, February 28th, at the Hospital.

Royal Free Hospital.—Annual Meeting at half past four p.m., on Wednesday, February 28th, at the Hospital.

National Orthopædic Hospital.—Annual Meeting on Wednesday, February 28th, at the Hospital.

Royal National Hospital for Consumption, Ventnor.—Annual Meeting at four p.m., on Wednesday, February 28th, at 34, Craven Street, Charing Cross.

Queen Charlotte's Hospital.—Annual Meeting at half-past four p.m., on Monday, February 26th, at the Hospital.

THE Prince of Wales has promised, as representing the Queen, to open the new Royal Infirmary at Newcastle during June.

MR. F. J. STEWARD, M.S., F.R.C.S., has been appointed an assistant surgeon to Guy's Hospital; also as assistant surgeon to the Hospital for Sick Children, Great Ormond Street.

THE Local Government Board are issuing an order to the Unions of Liverpool, West Derby, and Toxteth Park to combine and provide an infirmary for tuberculous paupers. A joint committee will be appointed, and any disputes are to be referred to the Local Government Board, whose decision is to be held final.

IT is satisfactory to note that the Irish Local Government Board are to have the services of an official bacteriologist. Professor E. J. McWeeney, who has for several years occupied the chair of Bacteriology and Pathology at the medical school of the Catholic University, Dublin, has been appointed to the office.

IT is quite a novel and welcome change to find the profits through the war urged in an appeal to public generosity. Such was the case the other day at Liverpool, when the Mayor, speaking at a meeting of the Royal Southern Hospital, made an appeal to the rich for that and other charities of the city, and stated that £50,000 would put the institution in funds, and some special effort might well be made, seeing that he knew instances where as much as £200,000 had been reaped through the war.

THE Local Government Board have consented that the Yardley Road Hospital at Birmingham shall be reserved for typhoid and diphtheria cases if some institution can be found to receive any small-pox cases that, but for the change, would have been allotted to the Yardley Road Hospital.

SURGEON-GENERAL W. TAYLOR, M.D., C.B., who has just been made the recipient of a good service pension, has had a distinguished career and most varied experiences. He is principal medical officer on the staff of the Commander-in-Chief in India, and has served 35 years in the army, and been on active service seven times. He received the C.B. for his services in the Khartoum Expedition in 1898, and the Order of the Medjidie at the same time. He has numerous decorations, and has been mentioned three times in dispatches.

THE experiment of receiving consumptive patients and carrying out the outdoor treatment as far as circumstances would permit, has been tried at the Sheffield Royal Infirmary. The climate of Sheffield is not a favourable one, nor were the arrangements specially adapted for the cases. Everything that careful treatment could do was done, but the result goes to show that locality and specially arranged buildings are large factors in securing satisfactory results. This should encourage those who are desirous of establishing sanatoria in climates indicated for the phthisical, that they are securing for those afflicted advantages which they could not secure without such establishments.

THE Syracuse Convalescent Home for Wounded Soldiers at Torquay has been opened this month. The home is beautifully situated, and can now receive 30 inmates. It has been placed in the hands of the committee rent free by Mr. Lee, and it has been decorated by him throughout. The War Office has sanctioned the scheme, and £2,350 has been contributed towards its maintenance. In addition, so many gifts have been sent to the home that only a small sum had to be spent in furnishing. The Cornwall wing will be maintained by Mr. and Mrs. Lagland-Barratt. Lord Lansdowne has sent a letter thanking the people of Torquay for their patriotic effort.

SOME time ago we reported that Dr. Cullen, medical officer of health for the Castlenock District of Dublin, had been asked to resign by the Local Government Board. Since then, owing to a petition against this proceeding by the North Dublin Union, another investigation has been held. The symptoms of an alarming laxity in notification by the local medical men was the chief outcome of the inquiry. Dr. Cullen gave evidence of the prevailing custom to exonerate himself from the charge of exceptional neglect. The surprising revelations of the inquiry caused the Council of the Dublin Sanitary Association to pass a strong resolution condemning the lax practices respecting notification adopted by the Dublin physicians. In his presidential address to the Dublin Branch of the British Medical Association, Sir Charles Cameron, medical superintendent officer of health for the same city, admitted, whilst regretting, the common omission of notification by medical men, and said many were even opposed to it, whilst others adhering to the law lost popularity amongst their patients.

